# Endosymbionts affect plant virus transmission by winged and wingless aphids

**DOI:** 10.1093/ismeco/ycag096

**Published:** 2026-04-25

**Authors:** Patricia Sanches, Mark C Mescher, Consuelo M De Moraes

**Affiliations:** Department of Environmental Systems Science, ETH Zürich, 8092 Zürich, Switzerland; Department of Environmental Systems Science, ETH Zürich, 8092 Zürich, Switzerland; Department of Environmental Systems Science, ETH Zürich, 8092 Zürich, Switzerland

**Keywords:** symbiosis, pathogens, phenotypic plasticity, vector-borne diseases, microbial ecology, virology

## Abstract

Vector-borne pathogens frequently modify host–vector interactions, and their influence can be modulated by other microbial symbionts. We recently documented endosymbiont effects on aphid traits involved in plant virus transmission, showing that facultative endobacteria—particularly *Hamiltonella defensa*—enhanced transmission of pea enation mosaic virus. Here, we examine transmission steps and associated molecular signatures in winged and wingless aphid morphs. Consistent with our previous findings, we observed enhanced pea enation mosaic virus transmission, as well as elevated viral titer in wingless aphids harboring *H. defensa*. However, winged aphids with this endosymbiont displayed similar effects on virus titer but not transmission. Furthermore, whereas wingless aphids exhibited higher transmission than winged aphids when *H. defensa* was present, this pattern was reversed for aphids harboring only the obligate endosymbiont *Buchnera aphidicola*; in parallel, we observed no differences between morphs of lines harboring other facultative endosymbionts. Subsequent experiments comparing lines harboring *H. defensa* versus the obligate symbiont alone revealed divergent effects on winged and wingless morphs on (*i*) virus inoculation efficiency (i.e., delivery of acquired virus; *H. defensa*), (*ii*) key salivary proteins (carbonic anhydrases, *CA*s; both lines), and (*iii*) plant defense-related marker transcripts (*PR-1*, salicylic acid pathway; *LOX*, jasmonic acid pathway; both lines). The correspondence of these patterns to the observed transmission effects suggests that endosymbiont-mediated effects on transmission may reflect changes in salivary secretions and related feeding traits. Our findings highlight the role of vector endosymbionts in disease transmission and provide insights into candidate processes by which they may influence virus–vector–host interactions.

## Graphical Abstract

**Graphical Abstract:**
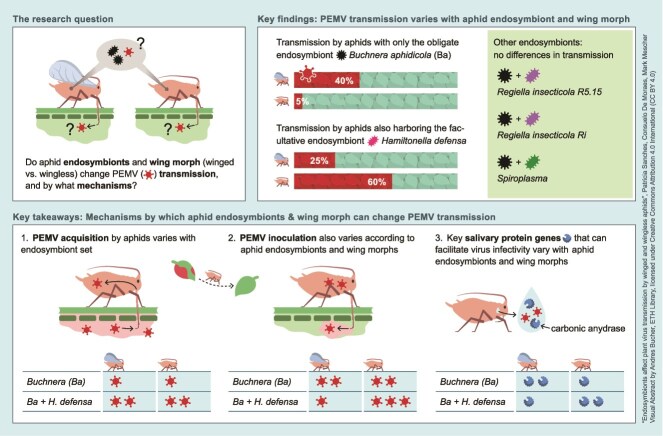

## Introduction

Microbial symbionts can have profound effects on traits of their multicellular hosts and host interactions with other organisms [1]. In the context of pathogen transmission, this has motivated a new focus on research at the interface of microbial and disease ecology [2–5]. For example, there is now considerable evidence that plant viruses can modify traits of both host plants and vector insects in ways that influence interactions between them, often in ways that enhance transmission [6–9]. Less work has explored how such effects are influenced by the presence of other microbes, though symbionts associated with both host plants and insect vectors appear to modulate virus effects on plant–insect interactions [4, 10–13]. To date, however, the mechanisms underlying such effects are not well understood [2, 3, 5].

Aphids transmit a third of vector-borne plant viruses [14, 15] and also have well-studied associations with endosymbiotic bacteria, including obligate endosymbionts (typically *Buchnera aphidicola*) that supplement essential amino acids and vitamins limited in the phloem diet [16]. Most aphids also harbor facultative endosymbionts, which can complement the metabolomic functions of the obligate endosymbiont and influence diverse traits [15, 17]. Facultative endobacteria can modulate aphid–plant interactions by influencing traits such as fecundity, feeding behavior, and the production of winged dispersal morphs [4, 18–20]. Such effects have context-dependent benefits and costs, which can influence endosymbiont persistence within aphid populations [15, 17, 19, 20]. Given their central role in aphid biology and ecology, endosymbionts are well placed to influence aphid-borne virus transmission [21, 22] and limited evidence documents endosymbiont effects on transmission rates [4, 23, 24]; however, the scope of such effects remain uncertain.

Plant virus transmission depends on aphid interactions with both infected and uninfected host plants, and there is considerable evidence that viruses influence these interactions by modifying vector and host traits [8, 9, 25]. For example, aphid-borne viruses can alter host-plant defense mechanisms and nutritional composition, as well as plant volatile emissions and visual cues that enhance vector attraction [6, 7, 26, 27]. They can also directly affect the host-seeking behaviors and sensory preferences of infected aphid vectors and thereby facilitate transmission to novel hosts [28, 29]. Emerging evidence also suggests that such effects are influenced by the presence of other microbial symbionts associated with hosts [10–13] or vectors [4, 24].

Recently, we reported that endobacteria can modulate aphid traits and aphid–plant interactions in ways relevant to plant virus transmission [4]. Using genetically uniform pea aphid lines (*Acyrthosiphon pisum* clone LSR1) associated with different endosymbionts, we found that certain facultative endobacteria, particularly *Hamiltonella defensa* and *Regiella insecticola* strain Ri, altered aphid traits in ways predicted to enhance the transmission of pea enation mosaic virus (PEMV; a persistently transmitted, circulative non-propagative virus). These effects included increased reproduction on virus-infected plants and behavioral preferences for host plants with infection status opposite that of the aphid vector [4]. We also observed elevated rates of virus transmission by individuals harboring *H. defensa* [4] even in experiments narrowly focused on aphid feeding, which excluded potential effects on dispersal and host-plant selection [30]. We also documented strong effects of virus–endosymbiont interactions on aphid metabolomic profiles [4], although the precise mechanisms underlying the observed transmission effects remain uncertain.

Because persistently transmitted viruses reside or circulate within vector tissues, their fitness depends critically on adaptation to the internal environment provided by the aphid [25, 31], including the presence of endobacteria [4, 24]. Endosymbionts may influence key transmission processes, such as virus acquisition, retention, or inoculation [8, 9], potentially leading to endosymbiont-specific effects on virus spread [2, 3, 5]. Although little research has examined how facultative endobacteria influence aphid-vectored virus transmission [2, 5], studies in whiteflies and mosquitoes have reported effects on virus uptake, circulation in the hemolymph, and storage in salivary glands [32–34]. Endosymbionts may also modulate feeding behaviors and have been shown to influence aphids’ host-plant preferences and probing behavior [4, 23]. Furthermore, endosymbionts can influence post-inoculation processes crucial for pathogen spread within host plants. For example, facultative aphid endosymbionts can affect levels of key aphid salivary proteins, such as histidine-rich Ca^2+^-binding protein (*HRC*) [35]. Other studies show that salivary proteins, including *HRC* and carbonic anhydrases (*CA*s), modulate vesicle trafficking or regulate salicylic acid (SA) and jasmonic acid (JA) defense pathways, facilitating aphid feeding and increasing plant susceptibility to viral infection [35, 36]. Collectively, these findings suggest that microbial symbionts can influence multiple stages of virus transmission; yet, the extent of such effects remain to be elucidated. In particular, little or no research has explored the detailed effects of endosymbionts at key stages of the virus transmission cycle (e.g. acquisition, inoculation, and host infection) or their influence on critical determinants of infectivity, such as salivary protein regulation and host plant responses to aphid feeding.

Building on our previous research on virus-induced manipulation of aphid behavior and performance in wingless vectors [4], the current study instead investigates how aphid endosymbionts influence specific steps in aphid transmission of PEMV to fava bean plants and whether these effects differ between winged and wingless morphs. We assessed transmission rates using genetically uniform wingless pea aphid lines (LSR1) harboring different endosymbiont sets, but also examined clonal winged aphids, which play a key role in aphid and virus dispersal [37, 38]. For each wing morph, we used one aphid line associated solely with the obligate endosymbiont *B. aphidicola* and four others harboring *B. aphidicola* in combination with the facultative symbionts *H. defensa*, *R. insecticola* (strains Ri or R5.15), or *Spiroplasma* sp. Previous results indicate that winged aphids secrete higher levels of salivary proteins, potentially enhancing plant susceptibility to viruses [36]. In light of our finding that *H. defensa* increases PEMV transmission in wingless aphids [4], we therefore hypothesized that wingless aphids harboring *H. defensa* would show elevated virus acquisition and inoculation, increased levels of salivary proteins, and reduced abundance of plant defense-related marker transcripts. We also predicted that winged aphids—regardless of endosymbiont composition—would exhibit enhanced transmission efficiency, salivary protein levels, and reduced abundance of plant defense-related marker transcripts relative to wingless morphs. To test these hypotheses, we analyzed transmission relevant factors such as virus acquisition and inoculation by aphid vectors, as well as transcript abundance of key aphid salivary proteins (*CA-II*, *CA-VII*, and *HRC* [35, 36]). We also investigated plant defense-related marker transcripts, including abundance of pathogenesis-related protein 1 (*PR-1*) in the SA pathway and Lipoxygenase (*LOX*) in the JA pathway, both of which have previously been used as plant response markers in PEMV pathosystems [31, 39]. Our results indicate that aphid endosymbionts modulate stepwise, morph-specific differences in transmission-relevant traits, thereby influencing plant virus transmission and shedding light on the molecular and ecological underpinnings of vector–pathogen interactions.

## Materials and methods

### Study system

Pea aphid (*A. pisum*) LSR1 clones harboring different endosymbionts were established as described previously [4]. Lines harbored *B. aphidicola* alone or with *R. insecticola* (Ri or R5.15), *Spiroplasma* (S161), or *H. defensa* (5A). Colonies were maintained on uninfected *Vicia faba* under controlled conditions (22°C, 60% RH, 16:8 h light:dark), with winged aphids induced by overcrowding. Experimental aphids were 9–12-day-old adults, and endosymbiont presence was confirmed by PCR [4].

Experimental plants were grown under the same conditions [4]. *V. faba* (var. *Fuego*) plants were grown individually in 9 × 9 × 10 cm pots containing potting substrate (Substrate 2; Klasmann-Deilmann GmbH, Germany) mixed with slow-release fertilizer (Osmocote Exact 5–6 M; NPK 15-9-12). Ten-day-old seedlings were PEMV- or mock-inoculated using aphids harboring only *B. aphidicola*, then kept aphid-free for four days. Infection was confirmed by ELISA 3 weeks later (Nano Diagnostics, AR, USA), 1 week prior to bioassays.

### PEMV transmission assay

To assess endosymbiont effects on PEMV transmission, pairs of wingless and winged aphids were placed on PEMV-infected plants for 48 h with access to the entire plant (per aphid line: 10 plants; two wingless and two winged aphids per plant). Each aphid was then transferred individually to a 10-day-old fava bean seedling and allowed to feed for 48 h. Virus-free, aphid-free control plants were processed in parallel. Seven days post-inoculation, plants were harvested and freeze-dried for molecular analysis. Aphids were collected in 1.5 ml tubes and freeze-dried. A random subset of aphids was analyzed for PEMV depending on whether their recipient plants were infected or uninfected (*N* = 4 per endosymbiont set, wing status, and plant outcome).

### PEMV acquisition by aphids

To explore effects on virus acquisition, PEMV abundance was quantified in aphids immediately after a 48 h acquisition access period. Pairs of wingless and winged aphids were placed on a PEMV-infected plant for 48 h, then collected for molecular analysis.

### PEMV inoculation by aphids

Because acquisition rates did not fully explain transmission patterns, we conducted additional experiments to assess other mechanisms, restricting subsequent work to aphids harboring only *B. aphidicola* or *B. aphidicola* + *H. defensa*. After a 48 h acquisition access period, 20 wingless and 20 winged aphids per endosymbiont set were transferred to feeding devices (9 cm Petri dishes) containing sterile tap water sealed with Parafilm, following previous methods [36] (per aphid line: three devices per wing morph). After 24 h, water samples were transferred to 50 ml tubes, freeze-dried, and analyzed for PEMV abundance.

### Aphids for measurement of salivary proteins

Because aphid salivary proteins can influence plant infectivity, we measured histidine-rich Ca^2+^-binding protein (*HRC*) and carbonic anhydrase (*CA*) *II* and *VII* in aphid vectors using methods similar to the virus acquisition experiment. Pairs of aphids were enclosed on uninfected or PEMV-infected plants for 48 h, collected in 1.5 ml Eppendorf tubes, and processed for molecular analyses. Parallel assays with virus-free aphids assessed baseline salivary protein levels and endosymbiont effects on aphid physiology (see Supplementary Information).

### Plant responses induced by aphids

Because plant defenses can influence aphid feeding and virus infection, we measured transcript abundance of pathogenesis-related protein 1 (*PR-1*; SA marker) and *LOX* (JA marker). After a 48 h acquisition access period on uninfected or PEMV-infected plants, groups of 10 wingless or winged aphids were transferred to 10-day-old seedlings and allowed to feed for 48 h (per aphid line: three plants per wing morph and virus condition). Half the plants were harvested immediately to quantify plant responses after two days of feeding (*N* = 3 per treatment), and the remainder were kept aphid-free for five additional days to mimic transmission assay conditions (*N* = 3 per treatment). For this assay, the entire aboveground portion of each plant was harvested, homogenized, freeze-dried, and processed for quantification of plant transcripts. Assays with virus-free aphids were also conducted to assess baseline plant responses across endosymbiont and wing-morph treatments (see Supplementary Information).

### Quantification of PEMV, salivary proteins, and plant responses

RNA was isolated from dried plant tissues, aphids, and feeding device samples for qRT-PCR quantification of PEMV, aphid salivary proteins, and plant response-related marker transcripts. PEMV RNA copy numbers in aphids (virus acquisition assays) and feeding devices were estimated using standard curves generated from in vitro–transcribed PEMV-1 RNA. Salivary protein and plant transcripts were quantified relative to aphid 28S rRNA and fava bean CYP2 genes, respectively, using the 2^−^ΔCt method [40, 41]. Reactions were run in technical duplicates on a StepOnePlus Real-Time PCR System (Applied Biosystems) using KAPA SYBR FAST qPCR mix (KAPA Biosystems). A Ct cutoff of ≤35 defined PEMV positivity in transmission, acquisition, and feeding device assays (see Supplementary Information; Supplementary Table S1 for primers).

### Statistical analysis

All analyses were performed in R 4.3.1 [42]. For all responses, we tested effects of endosymbiont, wing morph, and their interaction. Virus transmission (binary outcome) was analyzed with a binomial generalized linear mixed model (GLMM) with sub-replicate nested within replicate as a random effect (lme4 [43]). Virus acquisition by aphids and inoculation into feeding devices were quantified as standard curve–derived PEMV RNA copy numbers, log-transformed, and analyzed with Gaussian generalized linear models (GLMs) (stats [42]). For acquisition, Ct values for aphid 28S rRNA were included as a covariate to account for minor variation in RNA input and cDNA yield. All other responses were quantified by 2–ΔCt relative to plant CYP2 or aphid 28S rRNA, log2-transformed, and analyzed with Gaussian GLMs. Model assumptions were assessed with simulation-based residual diagnostics (DHARMa [44]). Fixed effects were evaluated using Type III Wald χ^2^ tests (car [45]), and post hoc comparisons used estimated marginal means with Tukey or Sidak correction (emmeans [46]). Effect sizes are reported as back-transformed values where applicable. Detailed methods and full model outputs are provided in the Supplementary Information (Supplementary Table S2).

## Results

### PEMV transmission by aphid vectors

**Figure 1:**
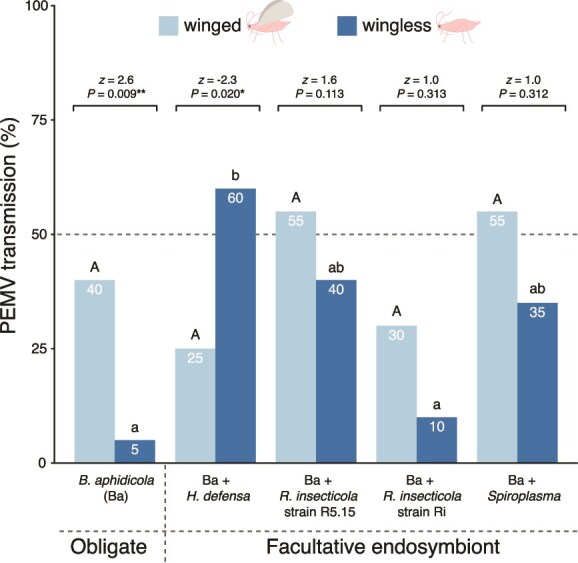
PEMV transmission by winged and wingless aphids harboring different endosymbionts. Numbers inside the bars indicate the percentage of plants that became infected with pea enation mosaic virus 1 (PEMV-1) for winged and wingless aphids associated with different endosymbiont sets. Generalized linear mixed model (*N* = 20). Uppercase letters indicate statistical differences across winged aphids, whereas lowercase letters indicate differences across wingless aphids according to multiple group comparisons (endosymbiont vs. wings) via Tukey’s test. Horizontal brackets represent comparisons between wingless and winged aphids harboring the same endosymbiont set (^**^P < .01, ^*^P < .05).

To examine endosymbiont effects on virus transmission, we placed virus-infected aphids on host plants for a controlled period and assessed plant infection rates—a design that isolates transmission effects associated with aphid feeding [4]. The results revealed strong interactive effects of aphid endosymbionts and wing status on virus transmission (GLMM, interaction: χ^2^ = 14.403, *P* = .006) (Fig. 1).

Endosymbiont effects were examined separately within each wing morph to determine morph-specific patterns. Consistent with our previous study [4], wingless aphids harboring *H. defensa* had the highest PEMV transmission rates, exceeding those harboring *R. insecticola* strain Ri (17-fold) or the obligate symbiont *B. aphidicola* alone (38-fold) (endosymbiont: χ^2^ = 14.293, *P* = .006) (Fig. 1). Transmission rates of wingless aphids harboring *R. insecticola* strain R5.15 or *Spiroplasma* did not differ from the other lines (*P* > .05) (Fig. 1). In contrast, in winged aphids, endosymbionts did not influence PEMV transmission rates (endosymbiont: χ^2^ = 6.053, *P* = .195) (Fig. 1).

The effect of wing morph was examined within each endosymbiont line. For aphids harboring only *B. aphidicola*, transmission was higher in winged than wingless aphids (19-fold, *P* = .009), whereas the opposite was observed for those additionally harboring *H. defensa* (5.4-fold in wingless than winged aphids, *P* = .02) (Fig. 1). Wing status did not affect transmission in aphid lines harboring other endosymbionts (*P* > .1) (Fig. 1).

These transmission patterns align with relative virus titers measured in host plants (Supplementary Fig. S1). Furthermore, we confirmed PEMV presence in aphids employed in the transmission assay (after the inoculation period), ruling out the absence of virions as an explanation for differential transmission (Supplementary Fig. S2).

### PEMV acquisition by aphid vectors

**Figure 2:**
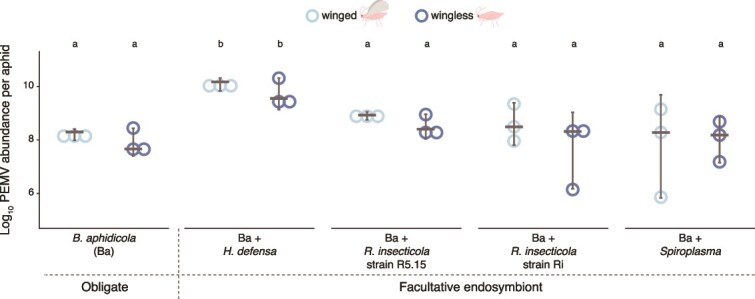
PEMV acquisition by winged and wingless aphids harboring different endosymbionts. Pea enation mosaic virus 1 (PEMV-1) abundance in pea aphids after a 48 h virus acquisition access period, measured in winged and wingless individuals associated with different endosymbiont sets. PEMV abundance was determined by amplifying a region of the PEMV-1 RdRp gene (spanning ORF1–ORF2) and normalized to the aphid 28S RNA gene. Dots represent PEMV-1 abundance individual aphids in each sample and horizontal grey lines are medians; error bars are 95% CIs. Generalized linear models (*N* = 3). Letters indicate statistical differences in PEMV-1 abundance according to multiple group comparisons (endosymbiont vs. wings) with Tukey’s test.

Although the infection status of aphids in our transmission assay was confirmed (Supplementary Fig. S2), variation in viral loads might still affect transmission. Therefore, we measured PEMV titers in aphids immediately after a controlled acquisition access period. These experiments revealed no interactive effects of wing status and endosymbionts (GLM, interaction: χ^2^ = 1.451, *P* = .834) (Fig. 2). Virus titers were instead influenced by endosymbionts alone (χ^2^ = 87.852, *P* = .001) and not by wing status (χ^2^ = 0.001, *P* > .9), with subsequent post hoc comparison indicating that aphids harboring *H. defensa* carried the highest virus abundance across all lines (>15-fold higher than other lines, *P* < .001), whereas virus abundance did not differ among the remaining symbiont lines (*P* > .05) (Fig. 2).

Endosymbiont effects were assessed separately for each wing morph, and we consistently found the highest virus acquisition by aphids harboring *H. defensa* for both wingless and winged morph (χ^2^ > 73.5, *P* < .001) (Fig. 2). Comparison of wing morph effects within each aphid-endosymbiont line revealed no differences in PEMV abundance (χ^2^ = 0.125, *P* = .723) (Fig. 2).

As in previous studies [47, 48], PEMV abundance did not increase over time in infected aphids maintained on plants that are non-hosts for PEMV, regardless of endosymbiont set or wing status, indicating that the viral load carried by aphids is determined by virus uptake during feeding (Supplementary Fig. S3).

### Post-acquisition mechanisms

As virus acquisition did not fully explain endosymbiont-mediated transmission patterns, particularly regarding differences between wing morphs within the same endosymbiont line, we next investigated post-acquisition mechanisms, including inoculation efficiency, salivary proteins involved in feeding, and induced plant responses. These assays focused on aphids harboring *B. aphidicola* alone or in combination with *H. defensa*.

#### Inoculation

**Figure 3:**
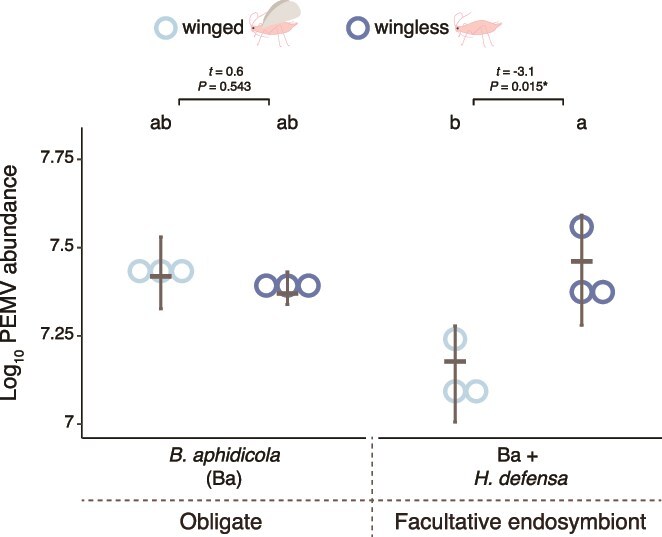
PEMV inoculation in feeding devices by winged and wingless aphids harboring different endosymbionts. Pea enation mosaic virus 1 (PEMV-1) in feeding devices after an inoculation access period of 24 h by wingless and winged aphids with different endosymbiont sets. PEMV abundance was determined by amplifying a region of the PEMV-1 RdRp gene (spanning ORF1–ORF2). Dots represent data from individual feeding devices and horizontal grey lines are medians; error bars are 95% CIs. Generalized linear model (*N* = 3). Letters indicate statistical differences in PEMV-1 abundance according to multiple group comparisons (endosymbiont vs. wings) with Tukey’s test. Horizontal brackets represent comparisons between wingless and winged aphids harboring the same endosymbiont set (^**^P < .01).

We assessed inoculation efficiency in water-filled feeding devices to exclude plant-mediated effects [36]. The results reveal strong interactive effects of endosymbionts and wing status on inoculated virus titers (GLM, interaction: χ^2^ = 6.980, *P* = .008), without effects of either endosymbiont or wing morph alone (χ^2^ < 3.1, *P* > .05) (Fig. 3). Post hoc comparisons showed that inoculated virus titers were two-fold higher in wingless than winged aphids harboring *H. defensa* (*P* = .015), whereas no differences were found between wing morphs for aphids harboring only *B. aphidicola* or between these morphs and those harboring *H. defensa* (*P* > .1) (Fig. 3). Overall, these patterns broadly align with the transmission differences observed in our previous assay for the line harboring *H. defensa*.

#### Salivary proteins

**Figure 4:**
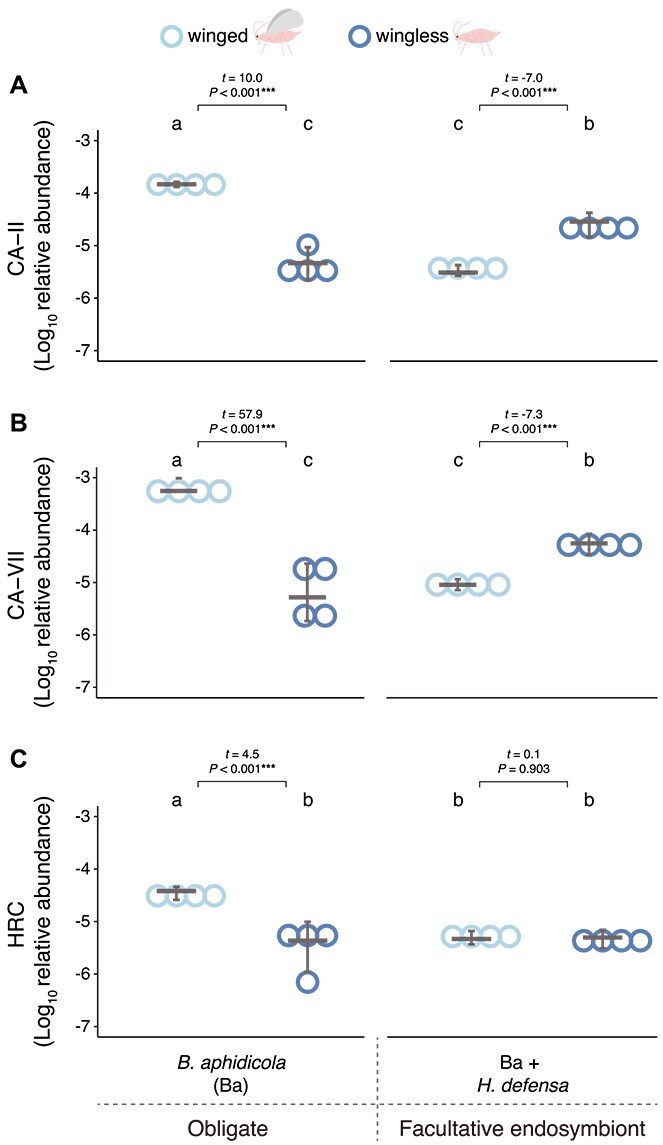
Salivary proteins levels in winged and wingless aphids vectoring PEMV and harboring different endosymbionts. (A) Levels of carbonic anhydrase II (*CA-II*), (B) carbonic anhydrase VII (*CA-VII*), and (C) histidine-rich Ca^2+^-binding (*HRC*) relative to aphid 28S RNA gene, measured in winged and wingless aphids vectoring pea enation mosaic virus (PEMV-1) and associated with different endosymbiont sets. Relative transcript abundance was normalized to the total number of aphids in each sample. Dots represent estimated transcript abundance for individual aphids in each sample and horizontal grey lines are medians; error bars are 95% CIs. Generalized linear models (*N* = 3). Letters indicate statistical differences in transcripts of salivary proteins genes according to multiple group comparisons (endosymbiont vs. wings) with Tukey’s test. Horizontal brackets represent comparisons between wingless and winged aphids harboring the same endosymbiont set (^***^P < .001).

Salivary protein transcripts in aphids were quantified after a controlled PEMV acquisition period, focusing on proteins previously shown to be influenced by endosymbionts or wing status, such as *CA-II*, *CA-VII*, and *HRC* [35, 36]. We found strong interactive effects of endosymbionts and wing morph on all three transcripts (GLM, interaction: *CA-II*: χ^2^ = 253.45, *P* = 2.2e–16; *CA-VII*: χ^2^ = 1204.6, *P* = 2.2e–16; *HRC*: χ^2^ = 13.059, *P* = .0003) (Fig. 4). Furthermore, post hoc multiple group comparisons revealed that *CA-II* and *CA-VII* were elevated in winged aphids harboring *B. aphidicola* (*CA-II*: five-fold and *CA-VII*: nine-fold higher than all other groups, *P* < .001), followed by levels in wingless aphids harboring *H. defensa* (*CA-II*: five-fold and *CA-VII*: 4.8-fold higher than *B. aphidicola* wingless) (Fig. 4A and B). The remaining aphid endosymbiont-wing treatments showed similarly low levels of these proteins (*P* > .1) (Fig. 4A and B). For *HRC*, post hoc comparisons indicated that winged aphids harboring only *B. aphidicola* had the highest levels (6.6-fold higher than all aphids; *P* = .037), whereas the remaining aphids had similar transcript levels (*P* > .1) (Fig. 4C).

Wing morph effects were assessed separately within each aphid line, and we found that both *CA-II* and *CA-VII* were elevated in wingless aphids compared to winged aphids harboring *H. defensa* (*CA-II*: 6.2-fold; *CA-VII*: 7.9-fold; *P* < .001), whereas the opposite pattern was observed in aphids harboring only the obligate endosymbiont *B. aphidicola* (*CA-II*: 26.9-fold; *CA-VII*: 46-fold; *P* < .001) (Fig. 4A and B). For *HRC*, transcript levels were also elevated in winged compared to wingless aphids harboring only *B. aphidicola* (10-fold; *P* = .037) but were not affected by wing status in aphids also harboring *H. defensa* (*P* = .75) (Fig. 4C).

Endosymbiont effects were examined separately within each wing morph. In winged aphids, all tested salivary proteins were elevated when aphids harbored *B. aphidicola* compared to *H. defensa* (*CA-II*: 42.5-fold; *CA-VII*: 59.8-fold; *HRC*: 6.6-fold; *P* < .001), whereas the reverse pattern was found for wingless aphids for both *CA-II* and *CA-VII* (*CA-II*: five-fold; *CA-VII*: 4.9-fold; *P* < .001) (Fig. 4A and B). No differences were observed for *HRC* in wingless aphids (*P* = .97) (Fig. 4C). These results are also broadly consistent with the transmission assay.

In virus-free aphids (analyzed independently from virus-infected ones; Supplementary Fig. S4), winged individuals harboring only *B. aphidicola* exhibited consistently elevated levels of all three salivary proteins. To evaluate how virus infection affected these patterns, we combined virus-free and virus-infected samples in a full model (endosymbiont vs. wing vs. infection status). This revealed that virus infection increased salivary protein transcripts in aphids harboring *B. aphidicola*, particularly in winged individuals (*CA-II*: 2.7-fold; *CA-VII*: 4.1-fold, *HRC*: 3.9-fold, *P* < .004). In aphids harboring *H. defensa*, virus infection had no detectable impact on *HRC* levels, but led to elevated levels of both *CA* proteins specifically in wingless individuals (*CA-II*: 3.5-fold, *CA-VII*: 4.1-fold, *P* < .002). These infection-dependent patterns were not observed in the virus-free analysis alone (Supplementary Fig. S4), indicating a specific virus–endosymbiont–morph interaction. Overall, these findings suggest that salivary proteins are normally elevated in winged aphids harboring only *B. aphidicola* and further enhanced by virus infection, whereas elevated levels of *CA* proteins is virus-dependent in wingless aphids harboring *H. defensa*.

#### Plant defense-related transcripts

**Figure 5:**
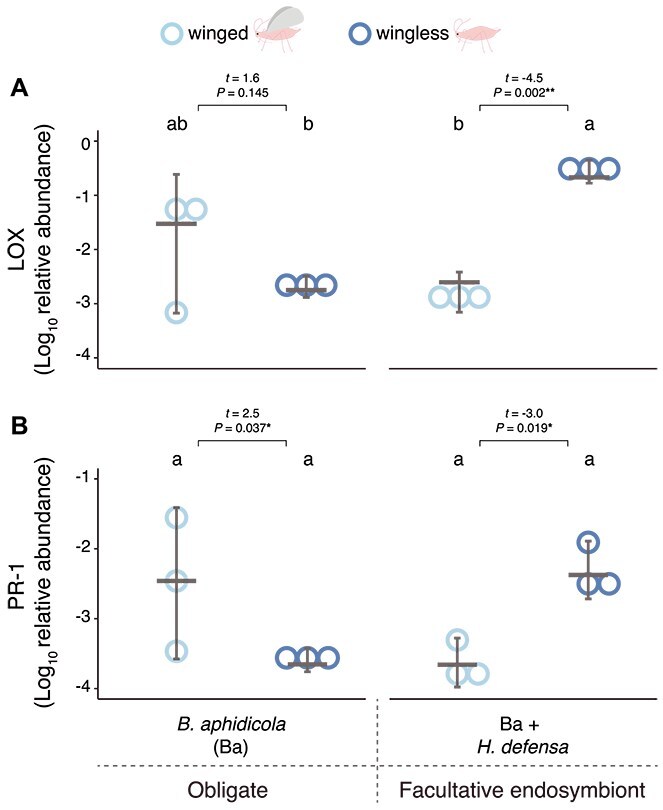
Plant defense-related marker transcript abundance after feeding by winged and wingless aphids vectoring PEMV and harboring different endosymbionts. (A) Levels of lipoxygenase (*LOX*) and (B) pathogenesis-related protein 1 (*PR-1*) in fava beans relative to *CYP2* gene (Cytochromes P450 family 2) immediately after 48 h feeding by winged and wingless aphids vectoring pea enation mosaic virus (PEMV-1) and associated with different endosymbiont sets. Dots represent transcript abundance for individual plants and horizontal grey lines are medians; error bars are 95% CIs. Generalized linear models (*N* = 3). Letters indicate statistical differences in plant defense-related transcript abundance according to multiple group comparisons (endosymbiont vs. wings) with Tukey’s test. Horizontal brackets represent comparisons between wingless and winged aphids harboring the same endosymbiont set (^**^P < .01, ^*^P < .05).

Because inoculation of plant viruses and salivary proteins by aphids can modulate plant responses that can influence feeding and infection outcomes, we examined *LOX* and *PR-1* transcript levels in plants immediately after feeding by PEMV-infected aphids. Our results revealed interactive effects of aphid endosymbionts and wing status on levels of both *LOX* and *PR-1* (GLMs, interaction on *LOX*: χ^2^ = 18.839, *P* = 1.422e–05; interaction on *PR-1*: χ^2^ = 15.094, *P* = .0001) (Fig. 5). Furthermore, post hoc multiple group comparisons revealed that *LOX* was elevated in wingless aphids harboring *H. defensa* (21-fold higher than all other groups; *P* < .001), followed by intermediate *LOX* abundance induced by winged aphids harboring only *B. aphidicola* (seven-fold than the remaining groups) (Fig. 5A). The remaining aphid lines induced similarly low *LOX* levels (*P* > .1) (Fig. 5A). For *PR-1*, post hoc comparisons indicated no differences across all combinations of aphid morphs and lines (*P* > .1) (Fig. 5B).

Wing morph effects were assessed separately within each aphid line. Comparisons within the line harboring only *B. aphidicola* revealed no effects of wing status on *LOX* levels (*P* > .1), whereas *PR-1* abundance was elevated following feeding by winged compared to wingless aphids (12-fold; *P* = .037) (Fig. 5). In contrast, within the aphid line harboring *H. defensa*, both *LOX* and *PR-1* were elevated following feeding by wingless compared to winged individuals (*LOX*: 167-fold; *PR-1*: 21-fold; *P* = .017) (Fig. 5).

Endosymbiont effects were examined separately within each wing morph, and we found that in winged aphids, *PR-1* transcript abundance was higher after feeding by aphids harboring only *B. aphidicola* compared to *H. defensa* (13.6-fold, *P* = .032), whereas there were no differences in *LOX* induction (*P* = .107) (Fig. 5). In wingless aphids, levels of both *LOX* and *PR-1* were higher after feeding by aphids harboring *H. defensa* compared to those harboring *B. aphidicola* (*LOX*: 135-fold, *PR-1*: 19-fold, *P* < .01) (Fig. 5). Overall, these results are also broadly consistent with the transmission assay.

To assess virus-independent effects, we performed a complementary analysis of plant responses induced only by virus-free aphid treatments (Supplementary Figs S5 and S6). In this analysis, plant transcripts were not influenced by wing status (*P* > .1), but responses were generally stronger when aphids harbored *H. defensa* compared to only *B. aphidicola* (2.3-fold; *P* = .028) (Supplementary Figs S5 and S6).

In a complementary assay, plants were first induced by aphid feeding for 2 days, followed by aphid removal to allow virus infection to develop for 5 more days—matching the conditions of our transmission assay. Except for reduced *LOX* levels in virus-infected plants after feeding by winged aphids harboring *B. aphidicola* (Fig. S6; *P* = .005), the abundance of plant transcripts remained largely unchanged, irrespective of endosymbiont composition, wing status, or virus presence (GLMs, all *P* > .1; Fig. S6).

## Discussion

The current results confirm our previous finding that the presence of the facultative endosymbiont *H. defensa* enhances transmission of PEMV by wingless aphid vectors (Fig. 1) [4]; moreover, we found similarly elevated rates of virus acquisition by these aphids (Fig. 2, Summary Supplementary Tables S3–S5). In winged aphids, however, increased virus uptake when *H. defensa* was present (Fig. 2) did not lead to higher transmission (Fig. 1), suggesting that differences in virus titer alone do not explain the observed transmission patterns (Supplementary Tables S3–S5). Further investigation revealed several patterns that are broadly consistent with transmission, specifically virus inoculation efficiency (Fig. 3), levels of *CA* salivary proteins (Fig. 4), and the abundance of the plant defense-related marker transcripts *LOX* and *PR-1* (Fig. 5, Supplementary Tables S3–S5). Together, these results highlight the influence of endosymbionts on aphid traits spanning various stages of the transmission process from virus acquisition to inoculation into new hosts, as well as plant responses to aphid feeding. These findings thus extend earlier work focused on behavioral and performance effects in wingless vectors [4] to a more mechanistically informed, morph-explicit framework for persistent virus transmission.

Transmission assays revealed that PEMV transmission was enhanced when wingless aphids harbored *H. defensa* (Fig. 1), adding to a small but growing body of research on microbial symbionts on insect-borne plant viruses [2, 4, 5, 24]. However, we found no evidence that *H. defensa* or other facultative endosymbionts affected PEMV transmission by winged vectors (Fig. 1), indicating that endosymbiont effects on plant virus transmission may be context-dependent and shaped by vector physiology. Moreover, we observed different endosymbiont-mediated effects in different wing morphs: aphids harboring *H. defensa* transmitted PEMV more effectively as wingless as winged, whereas aphids harboring only the obligate endosymbiont *B. aphidicola* exhibited the opposite pattern (Fig. 1). As our transmission and acquisition assays focused narrowly on effects during aphid feeding, they do not address other virus or endosymbiont effects on aphid traits and behaviors (e.g. dispersal and host–plant selection) that may also influence transmission [4]; nevertheless, they reveal effects on key steps in the transmission process.

Measurement of virus titers in aphid vectors revealed that endosymbionts significantly influence PEMV uptake (Fig. 2); yet, differences in virus acquisition do not fully explain transmission patterns (Fig. 1). Specifically, despite similar virus titers, transmission rates varied between wingless and winged aphids harboring the obligate endosymbiont alone or in combination with *H. defensa* (Fig. 2). Although little previous work has addressed either the effects of endosymbionts or wing status on virus uptake by aphids [36, 40], endosymbiont effects on virus acquisition have been documented in other pathosystems [2, 5, 49]. For example, *H. defensa* was reported to enhance the acquisition of several plant viruses by whitefly vectors [50–52], whereas the endobacteria *Serratia* sp. and *Wolbachia* were found to influence virus uptake by mosquitoes [34, 49]. Potential mechanisms underlying such effects include endosymbiont-secreted proteins that enhance gut membrane permeability to virions [34] or protect virions during circulation in the hemocoel [2, 5]. Although virus uptake plays a role in the transmission of persistently transmitted viruses like PEMV—which reside but do not replicate in aphid vectors [47, 48] (Fig. S3)—other factors such as virion storage in the salivary glands and subsequent inoculation into plant tissues may be key determinants of plant infection rates [8, 9, 53].

PEMV abundance in artificial feeding devices reflected endosymbiont-mediated effects on the efficiency of virus inoculation by aphids (Fig. 3). In contrast to virus acquisition (Figs 1 and 2), patterns of inoculation reflected the transmission patterns observed in wingless and winged vectors harboring *H. defensa* (Figs 1 and 3). Endosymbionts have previously been shown to influence aphid feeding behaviors, with implications for virus inoculation into host plants [4, 23, 54]. For example, *H. defensa* and *Arsenophonus* sp. were found to have opposing effects on aphid probing frequency [23]. The influence of wing status has been investigated in only one previous study, which focused on non-persistently transmitted viruses (which transiently attach to mouthparts) rather than persistent viruses like PEMV [31]. In that study, wingless and winged aphids exhibited similar feeding behaviors and virus inoculation rates in artificial feeding assays [36]. We might expect such effects to be more pronounced in persistently transmitted viruses, which face stronger selection for adaptation to vector traits [31]. In the current study, endosymbiont-mediated effects altered PEMV inoculation rates in aphids harboring *H. defensa* (Fig. 3), but non-significant differences between winged and wingless aphids harboring only the obligate symbiont did not reflect their marked transmission patterns (Fig. 1), suggesting that additional aphid-mediated mechanisms may also be important.

Aphid salivary proteins can directly mediate infection of host plats, even when virus inoculation titers are similar [36, 53], and our results reveal endosymbiont-mediated effects on salivary protein transcript abundance corresponding with transmission patterns (Figs 1 and 4, respectively). Previous research has shown that specific salivary proteins can enhance virus infectivity by facilitating intercellular movement within plant tissues. For example, cucumber mosaic and turnip mosaic viruses—both non-persistently transmitted and not phloem-limited—were highly infectious when transmitted by winged green peach aphids (*Myzus persicae*) via elevated *CA-II* salivary protein, which facilitated virus spread between cells [36]. Our findings show that salivary protein transcripts are also influenced by endosymbionts. Specifically, the facultative endosymbiont *H. defensa* appears to enhance salivary protein transcript levels and virus transmission in wingless but not in winged aphids (Figs 1, S1, and  4). Although *CA*s likely have minimal impact on systemic infection in phloem-limited viruses, PEMV may be an exception, as it can move from epidermal and mesophyll cells to the vasculature via a movement protein encoded by the virus’s second RNA component [47, 48, 55]. Further work is needed to clarify how PEMV interacts with the aphid transcriptome to enhance transmission, as our results suggest these effects may not extend to all salivary proteins, such as *HRC* (Fig. 4C). Whereas salivary protein levels are known to be regulated by physiological trade-offs between flying and settling morphs [36], their interplay with endosymbionts may more broadly influence aphid physiology [4], potentially affecting transcriptomic and proteomic regulation.

Endosymbiont-mediated effects on aphid feeding traits have been reported to attenuate plant responses [35, 56–58], but our measurements of *PR-1* and *LOX* showed no correlation between increased salivary protein transcript levels and reduced abundance of *LOX* and *PR-1* (Figs 4, 5, Supplementary  Figs  S4, and  S5). Such interactions may be more readily detectable through comprehensive investigations of induced plant pathways. Rather than a potential downregulation of responses, transcript levels mirrored patterns of plant recognition and defense responses triggered by both the pathogen and its vector [39]. In this context, *LOX* and *PR-1* serve as proxies for plant response marker transcripts, with *LOX* acting upstream in JA biosynthesis, rather than as direct quantitative readouts of these pathways. For example, virus inoculation (Fig. 3), transmission (Fig. 1), and salivary protein levels (Fig. 4) closely aligned with *LOX* and *PR-1* abundance (Fig. 5), suggesting these transcripts may reflect early recognition of aphid and virus cues rather than sustained resistance. Accordingly, *LOX* and *PR-1* levels returned to baseline in both uninfected and virus-infected plants after aphid removal (under conditions matching the transmission assay, Supplementary Fig. S6), suggesting that transient signaling, coupled with strong early inoculation and potential salivary protein input, may be sufficient to initiate infection before plant defenses can contain viral spread [59, 60].

Taken together, our results also have potential implications for disease dynamics, particularly at fine spatiotemporal scales. The appearance of winged individuals during the parthenogenetic phase, enabling dispersal, is critical for aphid survival under crowded or poor conditions [37]. Aphid-vectored pathogens also benefit from these dispersive traits—occasionally even inducing wing plasticity [38, 61]—which, may favor the evolution of traits mediating efficient transmission by winged aphids [25, 30, 36]. This expectation aligns with our findings showing consistent PEMV transmission by winged aphids with different endosymbionts (Fig. 1). In contrast, the fitness of wingless aphids relies heavily on prolific reproduction [37] and endosymbionts that enhance aphid performance on virus-infected plants—such as *H. defensa*—may increase in frequency, promoting local pathogen spread [62]. This may be one of several factors contributing to the higher PEMV transmission efficiency in wingless aphids harboring *H. defensa* (Fig. 1) [4], whereas the lower efficiency of aphids associated only with the obligate symbiont *B. aphidicola* (Fig. 1) might be explained by the reduced frequency of this aphid configuration in natural settings [63]. Improved understanding of such interactions may be critical for integrating within-population variation in aphid communities to spatiotemporal patterns of virus outbreaks [22, 63, 64].

The findings in this study provide proof-of-concept that facultative endosymbionts can modulate persistent plant virus transmission in a morph-specific context, based on a single pea aphid clone with well-characterized endosymbiont associations. However, accumulated mutations in long-held laboratory lines may limit the generality of our findings [65]. Given the strong influence of aphid genotype, endosymbiont strain, and virus isolate on ecological interactions, an important next step is to test whether the morph-by-endosymbiont patterns observed here extend across additional aphid genotypes, endosymbiont strains, and host races, in this and other pathosystems. At the same time, these results provide a foundation for mechanistic work. The clear endosymbiont-by-morph effects on transmission-relevant traits motivate deeper characterization of virus- and endosymbiont-mediated changes in transcriptomes and proteomes, followed by functional analyses in aphids and host plants, including assessment of downstream JA-responsive defense markers. More broadly, our results support more explicit integration of vector symbionts—and their morph-specific effects—into studies of vector-borne plant pathogens at the interface of microbial ecology and disease transmission.

In conclusion, our results demonstrate that endosymbionts mediate multiple mechanistic stages of virus transmission in a morph-specific manner. Specifically, enhanced PEMV transmission by wingless aphids harboring *H. defensa* is not explained by effects on virus acquisition but is consistent with observed endosymbiont effects on inoculation efficiency and *CA* salivary protein transcript abundance, which also were accompanied by similar plant patterns in *LOX* and *PR-1* transcript abundance. Moreover, we observed divergent transmission patterns between wingless and winged aphids harboring *H. defensa* compared to those associated only with the obligate endosymbiont, which also corresponded to differences in inoculation, salivary protein, and plant responses. These findings contribute to a growing body of research exploring how microbial symbionts shape pathogen transmission by modifying vector traits at behavioral and physiological levels and provide insights into candidate processes at the molecular levels [23, 32–34]. More broadly, these outcomes highlight the need to investigate the ecological and molecular processes underlying symbiont–virus–vector interactions and to explicitly incorporate endosymbiont- and morph-specific processes into studies of vector-borne plant pathogens.

## Supplementary Material

SI_Sanches_wings_endosymbionts_ycag096

## Data Availability

All data analyzed in this study are included in the manuscript and its supplementary information files. Source data and code are publicly available in the ETH Zürich repository (https://doi.org/10.3929/ethz-c-000790251).
