# Supplementary material for: Endosymbionts affect plant virus transmission by winged and wingless aphids
Source: ISME Commun. 2026 Apr 25;6(1):ycag096. doi: 10.1093/ismeco/ycag096 (PMC13155121; doi:10.1093/ismeco/ycag096)
Supplement: SI_Sanches_wings_endosymbionts_ycag096 [file si_sanches_wings_endosymbionts_ycag096.docx]

**Supplementary Information**

Endosymbionts affect plant virus transmission by winged and wingless aphids

Patricia Sanches^1^, Mark C. Mescher^1^, Consuelo M. De Moraes^1*^

^1^Department of Environmental Systems Science, ETH Zürich, Zürich, Switzerland

This file includes:

Extended Methods

Supplementary Figures S1-S6

Supplementary Tables S1-S5

**Extended Methods**

*PEMV retention by aphids*

In an additional analysis, we tested potential PEMV replication as well as PEMV retention over time in aphid vectors. Groups of five wingless aphids were enclosed with the cellophane bag in PEMV-infected plants for an acquisition access period of 48 h (three source plants per aphid line). After this period, a single aphid was sampled from each group (retention day 0), and the remaining aphids in the group were enclosed with the cellophane bag in a red clover plant (*Trifolium pratense*), which is a host for aphids but not for PEMV. After four days, we sampled another single aphid from each group (retention day 4). The same procedure was repeated in a separated assay testing winged aphids instead. Aphid samples were stored in a 1.5 ml Eppendorf tube, immediately flash-frozen in liquid nitrogen, lyophilized for 48 h, and then employed in molecular analysis to quantify PEMV levels. Tissues of red clovers were also used in molecular analysis to confirm the absence of PEMV.

*Quantification of PEMV, salivary proteins and plant defenses*

Dried plant tissues were transferred to 50 mL Falcon tubes and ground to a fine powder using three 3 mm glass beads in a Geno/Grinder® 2010 (SPEX® SamplePrep). Dried aphid samples were similarly processed in 1.5 mL Eppendorf tubes. Water samples from the feeding device inoculation assay were used directly for RNA isolation.

RNA extraction was carried out using TRI Reagent (Sigma-Aldrich), followed by chloroform separation and isopropanol precipitation. RNA pellets were washed with 80% ethanol, resuspended in DEPC-treated water (Sigma-Aldrich), and stored at −20 °C. Aphid and plant RNA samples were treated with DNase I (Thermo Fisher Scientific) and reverse transcribed using the RevertAid First Strand cDNA Synthesis Kit (Thermo Fisher Scientific), with a 1:1 mixture of oligo(dT) and random hexamer primers. In contrast, RNA from water samples (feeding devices) was used for cDNA synthesis without DNase treatment.

To quantify PEMV RNA abundance via absolute quantification, a region of the PEMV-1 RdRp gene (spanning ORF1–ORF2) was PCR-amplified from a highly infected plant cDNA sample using Phusion High-Fidelity DNA Polymerase (Thermo Fisher Scientific). The amplicon was purified using the GeneJET PCR Purification Kit (Thermo Fisher Scientific), cloned into the CloneJET PCR Cloning Vector (Thermo Fisher Scientific), and transformed into *E. coli* grown on LB medium with ampicillin. Colonies were screened via PCR and HindIII digestion, and a clone in the correct orientation was confirmed by Sanger sequencing. The plasmid was linearized with XbaI (Thermo Fisher Scientific) and used as template for in vitro transcription (HiScribe T7 Quick High Yield RNA Synthesis Kit, New England Biolabs). RNA transcripts were purified using phenol:chloroform extraction and quantified on an Agilent TapeStation.

A 5-fold serial dilution of the purified viral RNA was prepared starting at 5 ng/μL. Each dilution was mixed with total RNA extracted from virus-free aphids or plants, respectively, to simulate the amplification background and avoid overestimation of virus abundance during qPCR, following methods described previously [1]. These RNA dilutions underwent DNase treatment (when applicable), cDNA synthesis, and qRT-PCR using the same workflow as for experimental samples. RNA transcript concentration was then converted to absolute RNA copy number using the molecular weight of the transcript and Avogadro’s constant. Standard curves were generated by plotting Ct values against the log₁₀-transformed RNA copy numbers, with amplification efficiency calculated as described previously [1]. Curve efficiency was 95.5% for aphid samples and 89% for feeding device samples.

qRT-PCR was performed using the KAPA SYBR FAST qPCR Kit (KAPA Biosystems) on a StepOnePlus Real-Time PCR System (Applied Biosystems), including a melting curve step to verify amplification specificity. Reactions were run in technical duplicates, and primer sequences and thermocycling conditions are detailed in Table S1. For the *LOX* and *PR-1* markers, primers originally designed and validated in a previous studies were also empirically validated here in *Vicia faba*, yielding a single amplicon of the expected size and a single melting-curve peak, consistent with specific amplification of the corresponding *LOX* homologue in fava bean and with their previous use in related systems [2-3]. Prior to analysing gene expression data, we also validated endogenous reference genes for both aphids and plants: for aphids, a panel of candidate reference genes from previous work [4] was screened and 28S rRNA was identified as the most stable across endosymbiont and virus treatments, whereas for plants three commonly used reference genes [5] were tested and *CYP2* showed the most stable Ct values across endosymbiont, wing and virus conditions. The Ct distributions in our dataset further support the stability of these reference genes across treatments. A Ct threshold of ≤35 (based on the sensitivity of the standard curves) was applied to determine PEMV infection in aphid acquisition, feeding device, and transmission assays.

For PEMV quantification, absolute PEMV RNA copy number was estimated for each sample by interpolation against the respective standard curve. For aphid samples, PEMV RNA copy numbers are reported per pooled aphid sample (*N* = 2), without additional normalization to an aphid single-copy gene. For feeding devices, PEMV RNA copy number was estimated directly using the standard curve. Because PEMV does not replicate in aphids, we did not transform PEMV RNA copy numbers using the 2^–ΔCt method; instead, potential variation in aphid RNA input and cDNA yield was addressed by including aphid 28S rRNA Ct as a covariate in statistical models of virus acquisition (see Statistical Analysis – Expanded Details).

Salivary protein gene expression was quantified relative to the endogenous aphid 28S rRNA gene, while plant defense gene expression was quantified relative to the endogenous fava bean *CYP2* gene, both using the 2^–ΔCt method [6-7]. In addition, supplementary analyses including PEMV abundance in infected plant tissues (Fig. S1), presence of PEMV in aphids used in the transmission assay (Fig. S2), and PEMV abundance in aphids over time in the retention assay (Fig. S3) were quantified relative to endogenous reference genes using the 2^–ΔCt method [6], without standard curve interpolation (fava bean *CYP2* for plant samples and aphid 28S rRNA gene for aphid samples).

*Statistical Analysis - Expanded Details*

For each assay, we selected the statistical model structure based on the response distribution, study design, and residual diagnostics. Virus transmission (binary infection outcome; Fig. 1) was analyzed using a binomial GLMM with a nested random effect of sub-replicate within replicate, fitted with glmer() (lme4 [8]). Virus titers acquired by aphid vectors (Fig. 2) and inoculated into feeding devices (Fig. 3) were based on standard curve–derived copy numbers of PEMV RNA (see preceding section). These were log-transformed and analyzed using Gaussian GLMs, fitted with the glm() function (lme4 [8]; stats [9]). For virus acquisition by aphids, Ct values for the aphid 28S rRNA gene were additionally included as a covariate to account for minor variation in RNA input and cDNA yield among samples.

All remaining response variables were analyzed using relative quantification based on the endogenous plant or aphid *CYP2* and 28S rRNA genes, respectively, using the 2^–ΔCt method. These included aphid salivary protein expression (*CA-II*, *CA-VII*, HRC*;* Fig. 4, S4), plant defense gene expression (*PR-1*, *LOX*; Fig. 5, S5, S6), virus abundance in infected plants (Fig. S1), virus presence in aphids after the transmission assay (Fig. S2), and virus retention by aphids over time (Fig. S3). All were modeled as a function of endosymbiont, wing morph, and their interaction. Expression values were log-transformed prior to fitting Gaussian GLMs with glm() (stats [9]).

Fixed effects and their interactions were evaluated using Type II or Type III Wald χ² tests (Anova, car [10]). Model assumptions—including homoscedasticity, normality, and dispersion—were assessed using simulation-based residual diagnostics from the DHARMa package [11]. When significant interactions or fixed effects were detected, follow-up models were fitted within levels of one factor (e.g., endosymbiont or wing status) to clarify interaction effects. Pairwise post hoc comparisons were performed using emmeans() (emmeans [12]) with Tukey or Sidak correction for multiple testing. Post hoc tests were conducted for all significant main effects or interactions. Effect sizes are reported as back-transformed values where applicable. Additional model outputs summarizing effect sizes for three sets of comparisons is provided as follows: (i) wingless vs. winged morphs within each endosymbiont line (Table S3), (ii) wingless morphs compared to *Buchnera*-only aphids (Table S4), and (iii) winged morphs compared to *Buchnera*-only aphids (Table S5).

Full details of all statistical models and post hoc comparisons are available in the source data and code publicly available in the ETH Zurich repository (https://doi.org/10.3929/ethz-c-000790251).

**
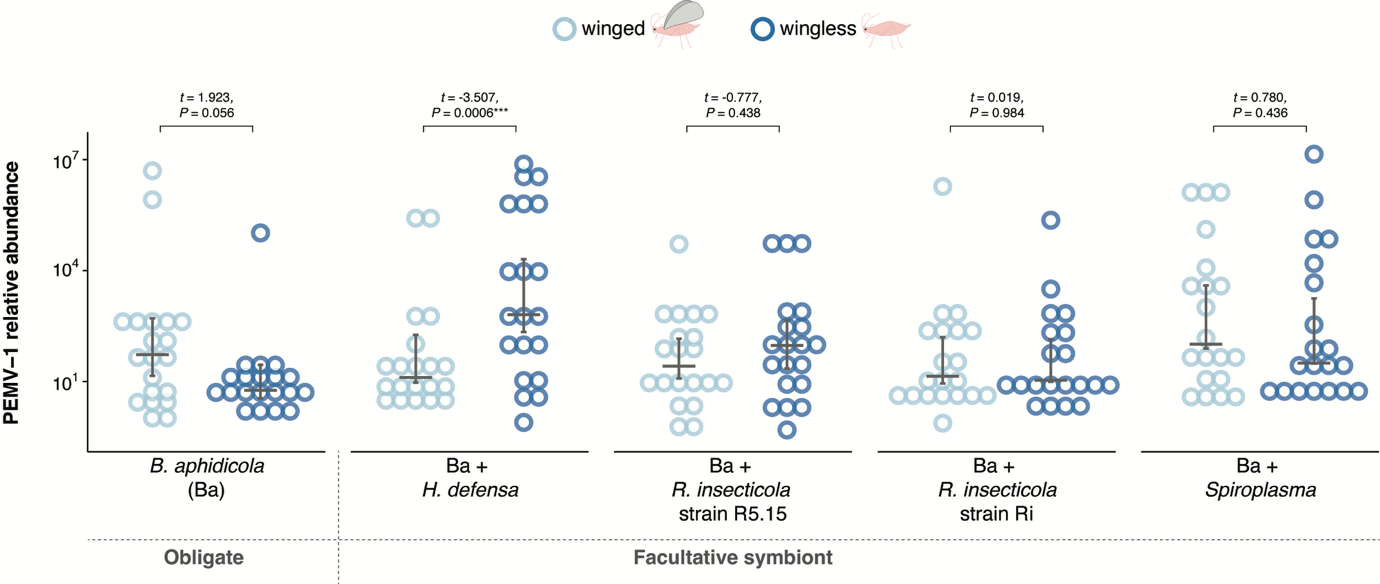
**

**Fig. S1: PEMV abundance in host plants after transmission by winged and wingless aphids harboring different endosymbionts.** Abundance of pea enation mosaic virus 1 transcript (PEMV-1) in fava bean plants relative to *CYP2* transcript (Cytochromes P450 family 2) after transmission by wingless and winged aphid vectors harboring different endosymbionts. Relative abundance values were normalized to values in the control plant condition (virus-free, aphid-free plants). Dots represent estimated abundance for individual plant samples and horizontal grey lines are medians; error bars are 95% CIs. Generalized linear mixed model (*N* = 20; interaction endosymbionts *vs.* wings: *X*^2^ = 16.725, *P* = 0.002; wings: *X*^2^ = 3.699, *P* = 0.054; endosymbionts: *X*^2^ = 8.314, *P* = 0.080). Horizontal brackets represent comparisons between wingless and winged aphids harboring the same endosymbiont set (****P* < 0.001).

**
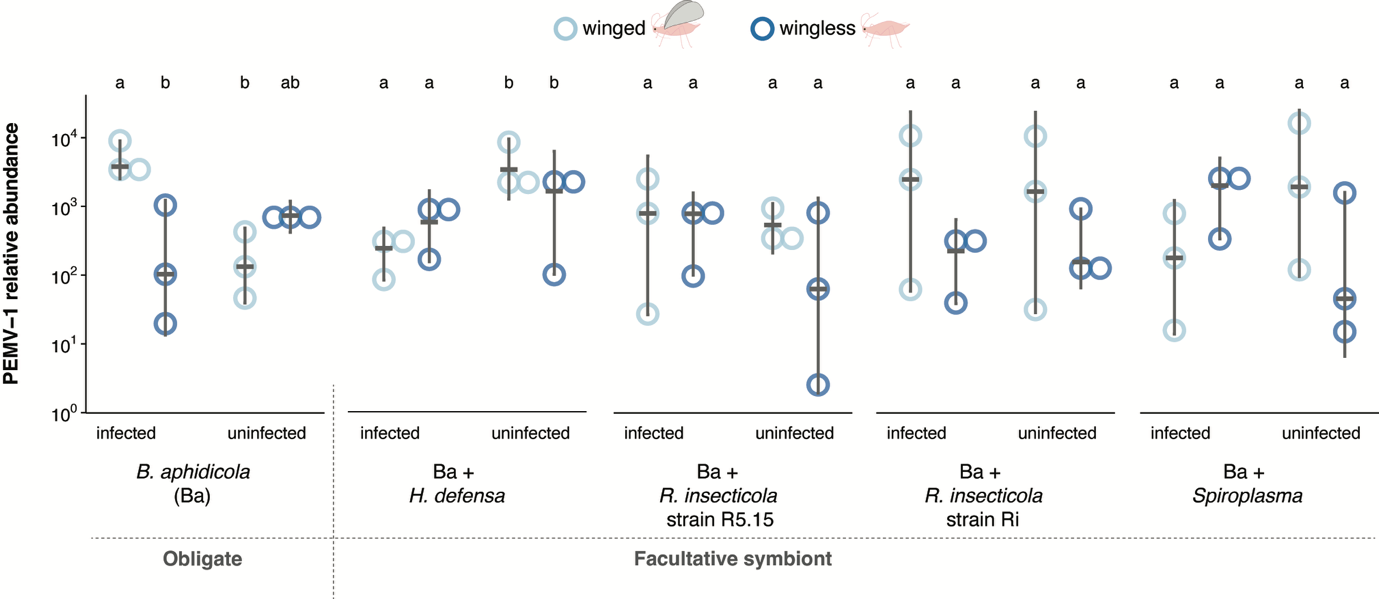
**

**Fig. S2: PEMV abundance in individual aphids after transmission assay.** Abundance of pea enation mosaic virus 1 transcript (PEMV-1) in pea aphids relative to aphid 28S RNA gene (*A. pisum*’s ribosomal RNA) after the virus transmission to fava beans, measured in winged and wingless individuals associated with different endosymbiont sets. Relative abundance values were normalized to abundance in virus-free wingless aphids from the control line (harboring only *B. aphidicola*). Dots represent abundance levels in individual aphids and horizontal grey lines are medians; error bars are 95% CIs. Generalized linear model (*N* = 3; interaction endosymbionts *vs*. wings *vs*. plant output: *X*^2^ = 14.585, *P* = 0.005). Letters indicate statistical differences in PEMV-1 expression within each aphid line according to multiple group comparisons (plant output vs. wings) with Tukey’s test.

**
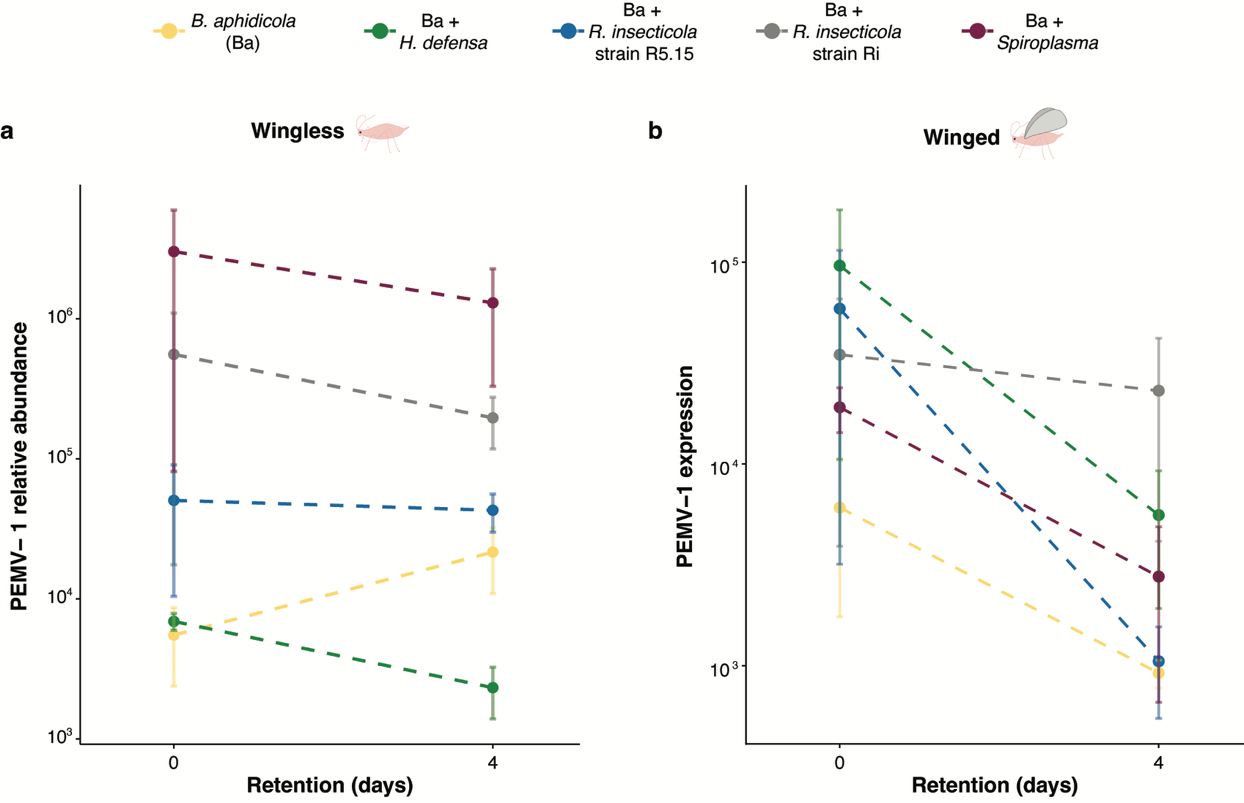
**

**Fig. S3: PEMV retention over time in winged and wingless aphids harboring different endosymbionts.** **(a)** Abundance of pea enation mosaic virus 1 transcript (PEMV-1) in wingless and **(b)** in winged aphids harboring different endosymbionts, measured after virus acquisition access period of 48 h (day 0) and after feeding for four days on red clover (*Trifolium pratense*; non-host plant for PEMV-1). PEMV-1 abundance in pea aphids were relative to aphid 28S RNA gene (*A. pisum*’s ribosomal RNA) and values were normalized to abundance in virus-free wingless aphids from the control line (harboring only *B. aphidicola*). Dots represent averaged abundance levels of samples containing two aphids and horizontal grey lines are medians; error bars are 95% CIs. Generalized linear models (*N* = 3; interaction endosymbionts *vs.* time, wingless: *X*^2^ = 2.054, *P* = 0.725, winged: *X*^2^ = 1.353, *P* = 0.852; effects of endosymbiont, wingless: *X*^2^ = 20.756, *P* = 0.003, winged: *X*^2^ = 5.847, *P* = 0.210; effects of time: wingless: *X*^2^ = 0.929, *P* = 0.335, winged: *X*^2^ = 3.996, *P* = 0.045; comparisons of abundance over time within each aphid line indicate no significant effects for both wingless and winged individuals (*t* < 1.572, *P* > 0.100).

**
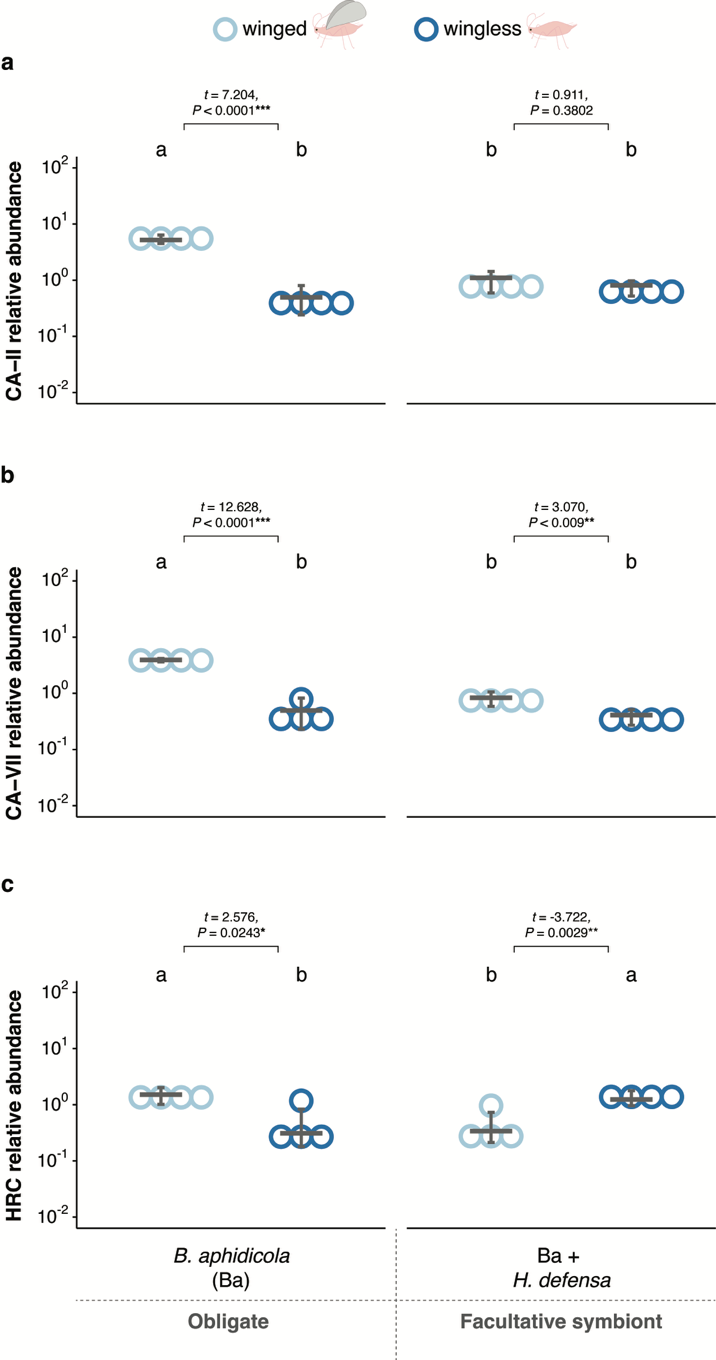
**

**Fig. S4: Salivary proteins levels in virus-free winged and wingless aphids harboring different endosymbionts.** **(a)** Levels of carbonic anhydrase II (*CA-II*), **(b)** carbonic anhydrase VII (*CA-VII*), and **(c)** histidine-rich Ca+2-binding (*HRC*) transcripts relative to aphid 28S RNA gene (*A. pisum*’s ribosomal RNA), measured in winged and wingless aphids vectoring pea enation mosaic virus 1 (PEMV-1) and associated with different endosymbiont sets. Relative abundance values were normalized to values in virus-free wingless aphids from the control line (harboring only *B. aphidicola*) and the total number of aphids in each sample. Dots represent estimated transcript abundance for individual aphids in each sample and horizontal grey lines are medians; error bars are 95% CIs. Generalized linear models (*N* = 3). Letters indicate statistical differences in salivary proteins transcript abundance according to multiple group comparisons (endosymbiont vs. wings) with Tukey’s test. Horizontal brackets represent comparisons between wingless and winged aphids harboring the same endosymbiont set (****P* < 0.001***P* < 0.01, **P* < 0.05).


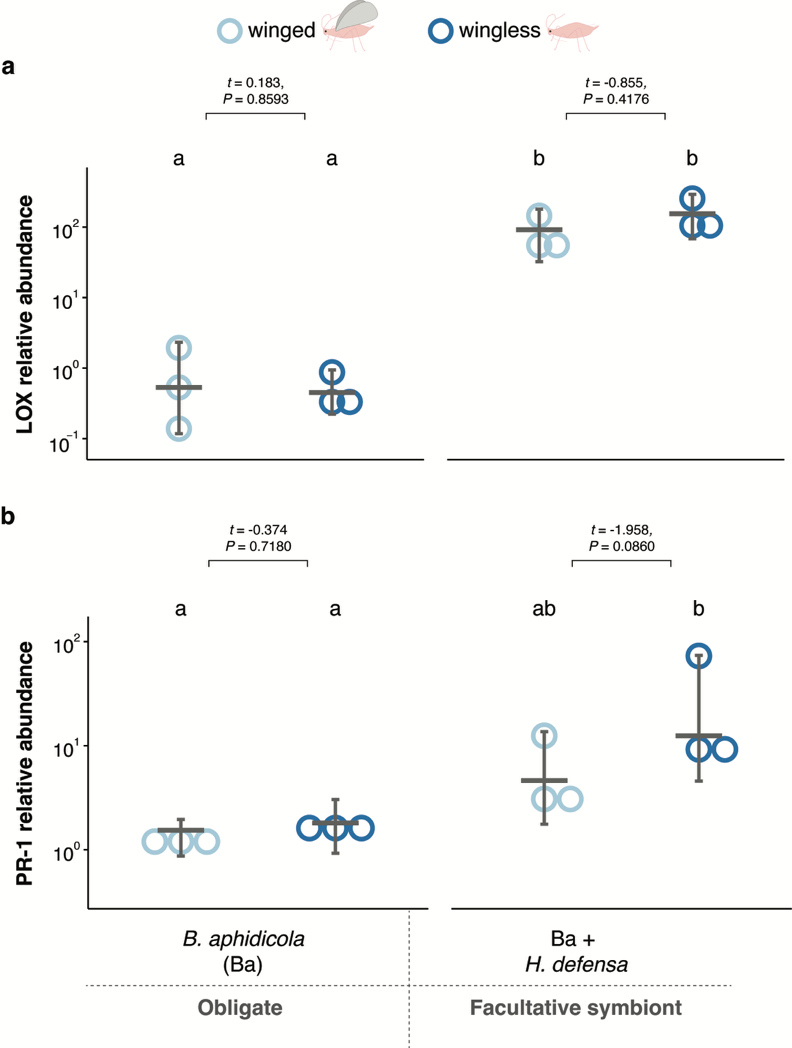


**Fig. S5: Abundance of plant defense-related transcripts after feeding by virus-free winged and wingless aphids harboring different endosymbionts.** **(a)** Levels of lipoxygenase (*LOX*) and **(b)** pathogenesis-related protein 1 (*PR-1*) transcripts in fava beans relative to CYP2 transcript (Cytochromes P450 family 2) immediately after 48 h feeding by winged and wingless aphids vectoring pea enation mosaic virus 1 (PEMV-1) and associated with different endosymbiont sets. Relative abundance values were normalized to values from control plants (virus-free and aphid-free plants). Dots represent values for individual plants and horizontal grey lines are medians; error bars are 95% CIs. Generalized linear models (*N* = 3). Letters indicate statistical differences in abundance of plant defense-related transcript according to multiple group comparisons (endosymbiont vs. wings) with Tukey’s test. Horizontal brackets represent comparisons between wingless and winged aphids harboring the same endosymbiont set.


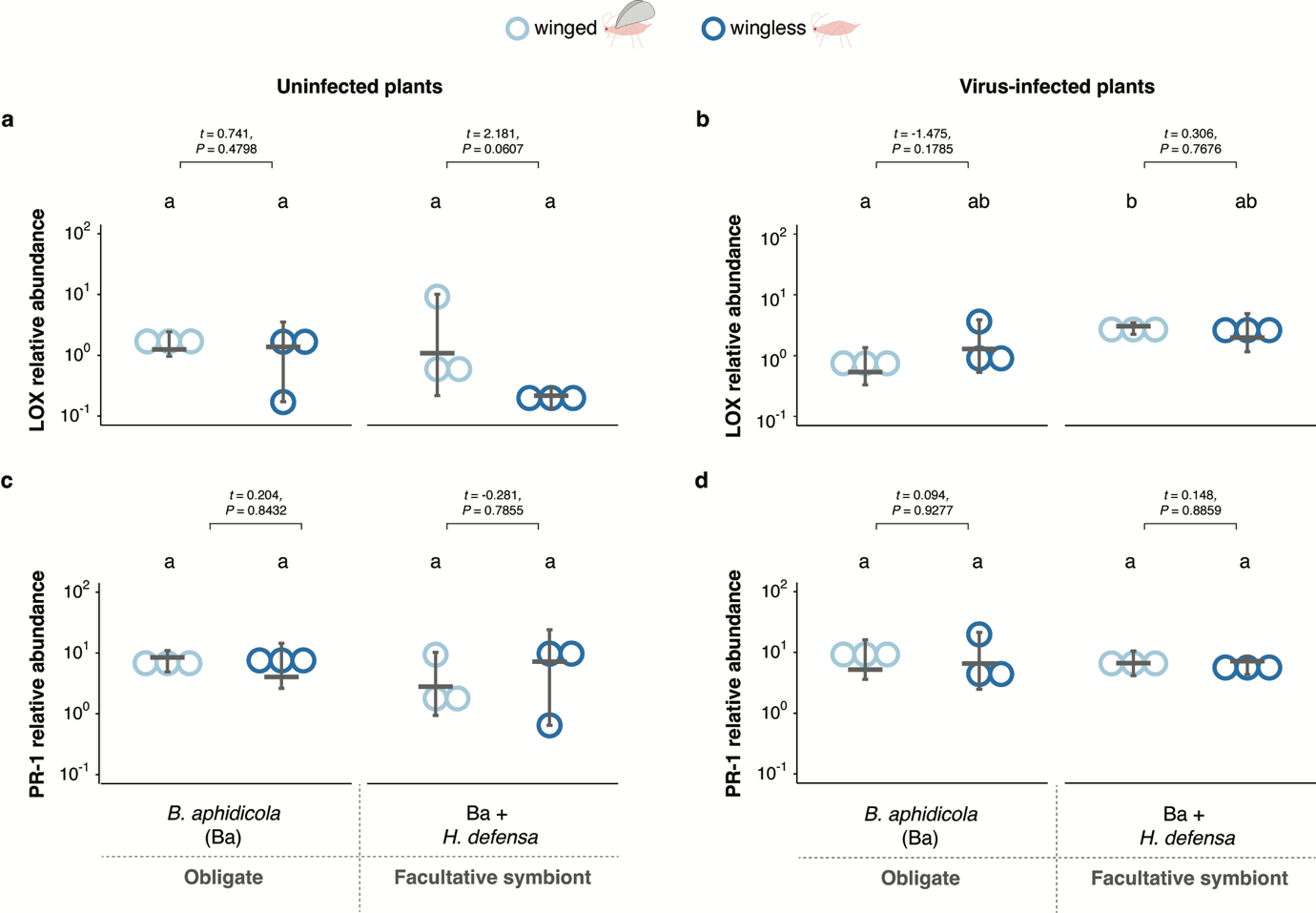


**Fig. S6: Abundance of plant defense-related transcripts five days after feeding by virus-free and PEMV-vectoring winged and wingless aphids harboring different endosymbionts. (a, c)** Levels of lipoxygenase (*LOX*) and pathogenesis-related protein 1 (*PR-1*) transcripts in fava beans relative to CYP2 transcript (Cytochromes P450 family 2) five days after feeding by virus-free aphids (uninfected plants), and **(b, d)** after feeding by aphids vectoring pea enation mosaic virus 1 (PEMV-1; virus-infected plants). Aphids were winged or wingless and harbored different endosymbiont sets. Relative abundance values were normalized to control plants (virus-free and aphid-free). Dots represent values for individual plants and horizontal grey lines are medians; error bars are 95% CIs. Generalized linear models (*N* = 3). Letters indicate statistical differences in abundance of plant defense-related transcript according to multiple group comparisons (endosymbiont vs. wings) with Tukey’s test. Horizontal brackets represent comparisons between wingless and winged aphids harboring the same endosymbiont set.

**Table S1.** Thermocycler conditions for quantification of plant virus, salivary proteins, and plant defenses.

| **Target** | | **Forward primer** | **Reverse primer** |
| --- | --- | --- | --- |
| *Vicia faba*  (reference gene) | CYP2 [5] | TGCCGATGTCACTCCCAGAA | CAGCGAACTTGGAACCGTAGA |
| *Acyrthosiphon pisum*  (reference gene) | 28S RNA gene [4] | CGGGTGGTAAACTCCATCTAAC | CGAGCGGTTTCACGTTCTTA |
| Pea enation mosaic virus 1 | PEMV-1 [13] | GCAATCCTACAGGACCTTCATA | CTCATCGTCTTCCGTGTCATC |
| Pea enation mosaic virus 1 - RdRp | PEMV-1 RdRp  (designed in this study) | ATTGTTTAGCATTGACTTGG | AATAAAGTCCTGGTTAAGGAG |
| Carbonic anhydrase II | CA-II  (designed in this study) | TCGAGATTAGCCAAGGAC | AAATTACCACCGGTTCCAC |
| Carbonic anhydrase VII | CA-VII  (designed in this study) | GGAGACAGTTAATGAAATTCAGAG | AATAACTAAGTACCAAGTGGCTAG |
| Histidine-rich Ca^+2^-binding | HRC [14] | TGGAAATACTTCCGGTCGGC | TTCTTCGGGGAACCTGAACG |
| Lipoxygenase | LOX [2] | AATTGATACCGCCACCTCTTT | CCAAGAATGCCTCTTTTGACA |
| Pathogenesis-related protein 1 | PR-1 [3] | CAGTGGTGACATAACAGGAGCAG | CATCCAACCCGAACCGAAT |

Cycling conditions

- PCR for PEMV-1 RdRp as target: 98°C 30 s, 35 cycles of (98°C 10 s, 57°C 20 s, 72°C 30 s), 72°C 10 min.
- qRT-PCR using all other targets: 95°C 3 min, 40 cycles of (95°C 15 s, 60°C 30 s, 72°C 30 s), 72°C 6 min. For melting curve analysis, a dissociation step cycle was added (55°C 10 s, 0.5°C for 10 s until 95°C).

**Table S2.** Overview of full model outputs from the main manuscript results testing the effects of endosymbiont, wing morph, and their interaction on virus abundance, aphid salivary protein transcripts, and plant response-related marker trnascripts. Values are from Type III ANOVA.

| **Figure** | **Assay** | **Model Type** | **Model Formula** | **Predictor: endosymbiont** | **Predictor: wing** | **Interaction: endosymbiont × wing** |
| --- | --- | --- | --- | --- | --- | --- |
| Fig. 1 | PEMV Transmission to Plants | GLMM (binomial) | infection ~ endosymbiont * wing + (1\|subrep/rep) | *Chisq* = 6.8;  *P* = 0.195 | *Chisq* = 6.6;  ***P* = 0.009** | *Chisq* = 14.4;  ***P* = 0.006** |
| Fig. 2 | PEMV Acquisition by Aphids | GLM (Gaussian) | PEMV ~ endosymbiont * wing | *Chisq* = 87.8;  ***P* < 0.001** | *Chisq* = 0.001;  *P* = 0.976 | *Chisq* = 1.4;  *P* = 0.834 |
| Fig. 3 | PEMV Inoculation by Aphids | GLM (Gaussian) | PEMV ~ endosymbiont * wing | *Chisq* = 2.0;  *P* = 0.152 | *Chisq* = 3.0;  *P* = 0.083 | *Chisq* = 6.9;  ***P* = 0.008** |
| Fig. 4a | Aphid Salivary Protein: *CA-II* | GLM (Gaussian) | CA-II ~ endosymbiont * wing | *Chisq* = 381.2;  ***P* < 0.001** | *Chisq* = 370.6;  ***P* < 0.001** | *Chisq* = 253.4;  ***P* < 0.001** |
| Fig. 4b | Aphid Salivary Protein: *CA-VII* | GLM (Gaussian) | CA-VII ~ endosymbiont * wing | *Chisq* = 2047.7;  ***P* < 0.001** | *Chisq* = 2027.9;  ***P* < 0.001** | *Chisq* = 1204.6;  P < 0.001 |
| Fig. 4c | Aphid Salivary Protein: *HRC* | GLM (Gaussian) | log(HRC) ~ endosymbiont * wing | *Chisq* = 18.9;  ***P* < 0.001** | *Chisq* = 27.5;  ***P* < 0.001** | *Chisq* = 13.1;  ***P* < 0.001** |
| Fig. 5a | Induced Plant Defense: *LOX* | GLM (Gaussian) | log(LOX) ~ endosymbiont * wing | *Chisq* = 3.2;  *P* = 0.069 | *Chisq* = 2.6;  *P* = 0.106 | *Chisq* = 18.8;  ***P* < 0.001** |
| Fig. 5b | Induced Plant Defense: *PR-1* | GLM (Gaussian) | log(PR1) ~ endosymbiont * wing | *Chisq* = 6.6;  ***P* = 0.010** | *Chisq* = 6.2;  ***P* = 0.012** | *Chisq* = 15.1;  ***P* < 0.001** |

**Table S3.** Summary of *within-line comparisons* between wingless and winged morphs across all measured traits. Values are fold changes (wingless relative to winged) from post hoc tests of full models including interaction terms; significance is indicated with asterisks based on Tukey or Sidak correction.

| **Endosymbiont** | **Transmission** | **Acquisition** | **Inoculation** | **Salivary proteins: *CA-II* and *CA-VII*** | **Pant defense: *LOX*** | **Pant defense: *PR-1*** |
| --- | --- | --- | --- | --- | --- | --- |
|  | **wingless *vs* winged** | **wingless *vs* winged** | **wingless *vs* winged** | **wingless *vs* winged** | **wingless *vs* winged** | **wingless *vs* winged** |
| *Buchnera aphidicola* (Ba) | ↓ 19x * | No differences between morphs | No differences between morphs | < ↓ 26x * | ↓ 6x | ↓ 12x * |
| Ba + *H. defensa* | ↑ 5.4x * | No differences between morphs | ↑ 2x * | > ↑ 6x * | ↑ 167x * | ↑ 21x * |
| Ba + *R. insecticola* strain R5.15 | No differences between morphs | No differences between morphs | Not assessed | | | |
| Ba + *R. insecticola* strain Ri | No differences between morphs | No differences between morphs |  |  |  |  |
| Ba + *Spiroplasma* | No differences between morphs | No differences between morphs |  |  |  |  |

**Table S4.** Summary of comparisons for each trait in *wingless aphids* using those harboring only the obligate endosymbiont Buchnera aphidicola as the baseline. Values are fold changes relative to this baseline from post hoc tests of full models including interaction terms; significance is indicated with asterisks based on Tukey or Sidak correction.

| **Endosymbiont** | **Transmission** | **Acquisition** | **Inoculation** | **Salivary proteins: *CA-II* and *CA-VII*** | **Pant defense: *LOX*** | **Pant defense: *PR-1*** |
| --- | --- | --- | --- | --- | --- | --- |
|  | **wingless** | **wingless** | **wingless** | **wingless** | **wingless** | **wingless** |
| *Buchnera aphidicola* (Ba) | baseline | baseline | baseline | baseline | baseline | baseline |
| Ba + *H. defensa* | ↑ 38x * | ↑ 74x * | ↑ 1.2x | > ↑ 4.5x * | ↑ 135x * | ↑ 19x * |
| Ba + *R. insecticola* strain R5.15 | ↑ 15x | ↑ 3.4x | Not assessed | | | |
| Ba + *R. insecticola* strain Ri | ↑ 2x | ↓ 1.6x |  |  |  |  |
| Ba + *Spiroplasma* | ↑ 15.3x | ↑ 1.2x |  |  |  |  |

**Table S5.** Summary of comparisons for each trait in *winged aphids* using those harboring only the obligate endosymbiont Buchnera aphidicola as the baseline. Values are fold changes relative to this baseline from post hoc tests of full models including interaction terms; significance is indicated with asterisks based on Tukey or Sidak correction.

| **Endosymbiont** | **Transmission** | **Acquisition** | **Inoculation** | **Salivary proteins: *CA-II* and *CA-VII*** | **Pant defense: *LOX*** | **Pant defense: *PR-1*** |
| --- | --- | --- | --- | --- | --- | --- |
|  | **winged** | **winged** | **winged** | **winged** | **winged** | **winged** |
| *Buchnera aphidicola* (Ba) | baseline | baseline | baseline | baseline | baseline | baseline |
| Ba + *H. defensa* | ↓ 2.7x | ↑ 117x * | ↓ 2x * | < ↓ 42x * | ↓ 7x | ↓ 13.6x * |
| Ba + *R. insecticola* strain R5.15 | ↑ 1.5x | ↑ 9.5x | Not assessed | | | |
| Ba + *R. insecticola* strain Ri | ↓ 2x | ↑ 2.8x |  |  |  |  |
| Ba + *Spiroplasma* | ↑ 1.5x | ↑ 2x |  |  |  |  |

**References**

1. Ruiz-Ruiz S, Moreno P, Guerri J, et al. A real-time RT-PCR assay for detection and absolute quantitation of Citrus tristeza virus in different plant tissues. *J Virol Methods* 2007; 145:96-105. [10.1016/j.jviromet.2007.05.011](https://doi.org/10.1016/j.jviromet.2007.05.011)
2. Braich S, Sudheesh S, Forster JW, et al. Characterisation of faba bean (*Vicia faba* L.) transcriptome using RNA-Seq: sequencing, de novo assembly, annotation, and expression analysis. *Agronomy* 2017; 7:53. [10.3390/agronomy7030053](https://doi.org/10.3390/agronomy7030053)
3. Rondoni G, Bertoldi V, Malek R, et al. *Vicia faba* plants respond to oviposition by invasive *Halyomorpha halys* activating direct defences against offspring. *J Pest Sci* 2018; 91:671–679. [10.1007/s10340-018-0955-3](https://doi.org/10.1007/s10340-018-0955-3)
4. Yang C, Pan H, Liu Y, et al. Selection of reference genes for expression analysis using quantitative real-time pcr in the pea aphid, *Acyrthosiphon pisum* (Harris) (Hemiptera, Aphidiae). *PLoS One* 2014; 9: e110454. [10.1371/journal.pone.0110454](https://doi.org/10.1371/journal.pone.0110454)
5. Gutierrez N, Giménez MJ, Palomino C, et al. Assessment of candidate reference genes for expression studies in *Vicia faba* L. by real-time quantitative PCR. *MOLB* 2011; 28: 13–24. [10.1007/s11032-010-9456-7](https://doi.org/10.1007/s11032-010-9456-7)
6. Livak KJ, Schmittgen TD. Analysis of relative gene expression data using real-time quantitative PCR and the 2− ΔΔCT method. Methods 2001; 25:402-408. [10.1006/meth.2001.1262](https://doi.org/10.1006/meth.2001.1262)
7. Higashi CH, Nichols WL, Chevignon G, et al. An aphid symbiont confers protection against a specialized RNA virus, another increases vulnerability to the same pathogen. Mol Ecol 2022;00:1–15. [10.1111/mec.16801](https://doi.org/10.1111/mec.16801)
8. Bates D, Mächler M, Bolker B, et al. Fitting Linear Mixed-Effects Models using lme4. *J Stat Softw* 2014;67. [10.18637/jss.v067.i01](https://doi.org/10.18637/jss.v067.i01)
9. Team RC. R: A language and environment for statistical computing. R Foundation for Statistical Computing, Vienna, Austria. *http://wwwR-project.org/* 2024.
10. Fox J, Weisberg S. *An R companion to applied regression*. Thousand Oaks: Sage Publications Inc, 2018.
11. Hartig F. DHARMa: Residual Diagnostics for Hierarchical (Multi-Level / Mixed) Regression Models. *R package* 2024; version 0.4.7. [cran.r-project.org/web/packages/DHARMa/vignettes/DHARMa.html](https://cran.r-project.org/web/packages/DHARMa/vignettes/DHARMa.html)
12. Lenth R, Banfai B, Bolker B, et al. Emmeans: Estimated marginal means, aka least-squares means. *R package version* 2022;1:3. [cran.r-project.org/package=emmeans](https://CRAN.R-project.org/package=emmeans)
13. Lee BW, Basu S, Bera S, et al. Responses to predation risk cues and alarm pheromones affect plant virus transmission by an aphid vector. *Oecologia* 2021; 196: 1005–1015. [10.1007/s00442-021-04989-6](https://doi.org/10.1007/s00442-021-04989-6)
14. Wang Q, Yuan E, Ling X, et al. An aphid facultative symbiont suppresses plant defence by manipulating aphid gene expression in salivary glands. *Plant Cell Environ* 2020; 43: 2311–2322. [10.1111/pce.13836](https://doi.org/10.1111/pce.13836)
